# Violencia entre parejas de la diversidad sexual y marcos teóricos explicativos: una agenda en desarrollo

**DOI:** 10.18294/sc.2025.5558

**Published:** 2025-04-15

**Authors:** Roberto Castro, Irene Casique Rodríguez

**Affiliations:** 1 PhD en Sociología Médica. Investigador titular, Centro Regional de Investigaciones Multidisciplinarias, Universidad Nacional Autónoma de México, Cuernavaca, México. lucia.ariza@gmail.com Universidad Nacional Autónoma de México Centro Regional de Investigaciones Multidisciplinarias Universidad Nacional Autónoma de México Cuernavaca Mexico lucia.ariza@gmail.com; 2 PhD en Sociología. Investigadora Titular, Centro Regional de Investigaciones Multidisciplinarias, Universidad Nacional Autónoma de México, Cuernavaca, México. lima.natacha@hotmail.com Universidad Nacional Autónoma de México Centro Regional de Investigaciones Multidisciplinarias Universidad Nacional Autónoma de México Cuernavaca Mexico lima.natacha@hotmail.com

**Keywords:** Violencia de Pareja, Minorías Sexuales y de Género, Feminismo, Construcción Social del Género

## Abstract

El objetivo de este artículo es explorar las teorías sociológicas existentes sobre la violencia entre parejas de la diversidad sexual para generar un marco explicativo e identificar los desafíos que enfrenta esta teorización en estas poblaciones. Tomamos como referencia el enfoque feminista y el enfoque de la violencia familiar vinculada a la violencia entre parejas heterosexuales, para analizar críticamente desde allí las nuevas teorizaciones que se han propuesto como marco explicativo de la violencia en parejas sexo diversas, particularmente, el feminismo posestructural y las teorías queer. Se observa que hay un creciente desarrollo de marcos teóricos e investigaciones empíricas enfocadas específicamente a explicar la violencia entre parejas LGBTIQ+. El artículo reflexiona sobre los elementos sociológico-feministas fundamentales que se deberían incorporar en las propuestas teóricas para explicar la violencia de parejas no heterosexuales y sobre algunos desafíos pendientes de resolver.

## INTRODUCCIÓN

En los últimos años se han producido importantes avances en torno a la investigación sobre la violencia de pareja, que ha sido reconocida como un problema de derechos humanos, político, social y de salud con muchas consecuencias para la vida de las personas^1^^,^^2^. La violencia íntima de pareja se define como

…todo comportamiento que, en una relación de pareja, causa daño físico, sexual o psicológico, incluidos los actos de agresión física, la coacción sexual, el maltrato psicológico y los comportamientos dominantes. Esta definición abarca la violencia infligida por los cónyuges y los compañeros actuales y anteriores.^3^

Sin embargo, la gran mayoría de los esfuerzos de investigación se han focalizado en las parejas heterosexuales, en detrimento de iniciativas para visibilizar y atender esta problemática cuando ocurre en parejas de la diversidad sexual^4^. Con la expresión “parejas de la diversidad sexual” (o parejas LGBTQ+) nos referimos a todas aquellas parejas que no corresponden a la descripción convencional de parejas “heterosexuales”. Esto incluye parejas en las que uno o ambos integrantes tienen identidad de género y/o orientación sexual no heteronormadas. Se trata, evidentemente, de un universo muy diverso que, por lo mismo, presenta dificultades importantes para su estudio. 

La narrativa dominante sobre la violencia de pareja se ha referido, por muchos años, a relaciones heterosexuales, en las que normalmente se identifica la figura de un agresor masculino y una víctima femenina^5^. Sin embargo, en los últimos años, el problema de la violencia en parejas LGBTIQ+ ha comenzado también a ser objeto de interés científico, inicialmente en el mundo anglosajón^6^, aunque poco a poco ha ido ganando espacio en la agenda investigativa de algunos países latinos como Chile y Puerto Rico. Ello se debe a la creciente evidencia de que la violencia en esas parejas es igual o mayor que en las parejas heterosexuales^7^. 

En América Latina, también ha comenzado a desarrollarse el interés por la violencia entre parejas LGBTIQ+ aunque siempre de manera marginal en comparación con la investigación (y el desarrollo de políticas públicas) sobre violencia entre parejas heterosexuales^8^. 

La teorización sobre la naturaleza, las causas y la dinámica de la violencia entre parejas LGBTIQ+ presenta un nivel de desarrollo menos consolidado que el alcanzado respecto a las parejas heterosexuales. Diversos autores postulan que la violencia entre parejas del mismo sexo no ha sido tan extensamente estudiada, entre otras razones, porque pone en entredicho el paradigma de género^9^^,^^10^. Otra razón es por la estigmatización que aún pesa en muchos países sobre las parejas del mismo sexo y, junto con ello, la preferencia de algunos grupos LGBTIQ+ de no llamar la atención sobre este tema para evitar una estigmatización adicional^11^^,^^12^^,^^13^, lo que además dificulta poder recurrir a la policía o a los servicios en caso de sufrir violencia^14^. Finalmente, mitos como la utopía lésbica (de relaciones más igualitarias entre dos mujeres) han sido señalados también como un factor que incide en una mayor normalización de las experiencias violentas en estas parejas y una mayor dificultad de reconocerlas como tal^15^. 

El objetivo de este artículo es explorar las teorías sociológicas existentes sobre la violencia entre parejas de la diversidad sexual para generar un marco explicativo e identificar los desafíos que enfrenta esta teorización en estas poblaciones. Para poner en contexto el tema, describimos los principales desarrollos teóricos sociológicos que se dieron en torno a la violencia entre parejas heterosexuales. A partir de ahí, nos centraremos en las críticas que se han hecho a estas teorías para abordar el tema en la población LGBTIQ+ y las nuevas teorizaciones que se han propuesto para explicar la violencia en parejas sexo diversas. Cabe aclarar que no es nuestra intención evaluar exhaustivamente el estado del arte ni proponer una nueva teoría sobre este tema, sino examinar, de manera crítica, las limitaciones de las propuestas teóricas que, con un enfoque sociológico y feminista, tradicionalmente han prevalecido en el estudio de la violencia en parejas heterosexuales y los retos que estas enfrentan como marco explicativo de la violencia en parejas de la diversidad sexual. En este sentido, intentamos identificar los elementos que, a partir de propuestas como el posestructuralismo y la sociología queer, son imprescindibles adoptar o incorporar en marcos teóricos más comprehensivos que permitan explicar mejor las complejidades de la violencia entre esas parejas.

## PARADIGMAS DOMINANTES

La violencia de pareja contra las mujeres ha sido investigada fundamentalmente desde dos escuelas o enfoques diferentes: el enfoque feminista y el enfoque de la violencia familiar. Tales abordajes presentan una diferencia fundamental: el enfoque feminista postula que la violencia contra las mujeres no es sino la versión extrema de un patrón estructural más general, que se refiere a la opresión de las mujeres en el sistema patriarcal, mientras el enfoque de la violencia familiar sostiene que el patrón más general que subyace a todas las formas de violencia familiar es la tolerancia cultural que nuestras sociedades tienen para con la violencia interpersonal. Estrechamente relacionada con esta diferencia de perspectivas se encuentra otra característica que distingue estos dos abordajes: el enfoque feminista ofrece una teorización más refinada a nivel macro, pero justo por eso enfrenta con frecuencia dificultades para operacionalizar y medir empíricamente conceptos claves como “patriarcado”^16^, “interseccionalidad”^17^ o “cultura de la violencia”^18^, por mencionar solo algunos. En contraste, el enfoque de la violencia familiar privilegia la investigación empírica, es decir, aquella basada en la operacionalización y medición de variables, y a partir de ahí propone teorías de alcance medio^19^, tales como la “teoría del ciclo de la violencia”^20^, la aplicación de la teoría del intercambio para explicar la violencia en la pareja^21^, la “teoría del estrés y el contexto social”^22^, o la “teoría de la violencia como control coercitivo”^23^, entre otros muchos ejemplos. De esta diversidad de perspectivas se derivan dos agendas científicas y políticas a veces diferentes, como examinaremos en seguida. 

### El enfoque feminista

Derivado de todo el trabajo hecho desde la década de 1970 para visibilizar y problematizar la violencia hacia las mujeres, se desarrolló la perspectiva feminista, que comprende el conjunto de teorías, prácticas y movimientos políticos que se centran en la lucha contra la opresión y discriminación de las mujeres. En el último cuarto del siglo pasado, desde el feminismo se postuló que la *causa última* de la violencia contra las mujeres (en todas sus formas, entre las que se incluye la violencia de pareja), es el patriarcado, es decir, el sistema social basado en la supremacía de los varones y lo masculino sobre las mujeres y lo femenino^24^^,^^25^. Esta *causa última*, sin embargo, dista de ser una explicación suficiente en un fenómeno como el que nos ocupa. 

La mayor parte de las investigaciones académicas y de las intervenciones de política pública que se hacen sobre la violencia de pareja en el mundo anglosajón y en América Latina, comenzaron su trayectoria bajo el impulso del paradigma feminista desde el activismo y la academia^26^. Las teorías feministas proporcionan un marco para explicar la violencia en la pareja, haciendo hincapié en las dinámicas de poder basadas en el género y la dominación masculina^27^. La narrativa predominante de esta perspectiva es que los hombres son los principales agresores y las mujeres las principales agredidas, y que la desigualdad entre hombres y mujeres está en el origen de la violencia de pareja y de la violencia contra las mujeres en general^24^^,^^25^^,^^28^.

En un comienzo, en la década de 1870, el problema se conocía predominantemente con términos como “violencia contra las esposas”^24^, “abuso de las esposas”^25^, o “golpizas a esposas”^29^. Sin embargo, como es propio en un campo con muchas disputas de poder^30^, los diversos actores en este debate fueron cambiando de jerarquía y de posición, lo que se tradujo en cambios en la denominación del problema: de las denominaciones anteriores se pasó a la violencia contra las mujeres, y de ahí a la violencia de género. Este último término empezó a generalizarse cuando los organismos internacionales de las Naciones Unidas se volvieron protagonistas en esta discusión y el concepto de género se institucionalizó^31^^,^^32^. 

Con el enfoque de género se postula que las diferencias biológicas entre los sexos no son las únicas que influyen en las desigualdades y discriminaciones que experimentan las personas. El concepto centra el análisis en cómo las construcciones sociales, culturales y políticas de género afectan a hombres y mujeres en diferentes formas y cómo estas desigualdades pueden estar en el origen de las diversas formas de violencia contra las mujeres. En cualquier caso, desde la perspectiva feminista se postula que la violencia de pareja contra las mujeres debe ser entendida como una forma más del conjunto de violencias que las mujeres sufren en diversos contextos, como la escuela, el trabajo o el espacio público, y que, por tanto, deben teorizarse como una expresión más de la desigualdad y opresión que las afecta.

Además de la dimensión estructural, algunas vertientes del feminismo han enfatizado la necesidad de centrar el análisis en el tipo de vínculo que se establece a nivel interpersonal entre las parejas. Así, en este plano, la violencia en parejas heterosexuales ha sido explicada por el uso que hacen los hombres de la violencia como un medio (entre otros) para ejercer control sobre las mujeres. Este uso de la violencia, a su vez, encuentra como fuentes de “legitimación” las normas patriarcales sobre las que se fundan las prerrogativas de los hombres en un contexto de clara desigualdad de géneros^25^^,^^26^^,^^33^.

Sin embargo, el papel del ejercicio del control como motivación de la violencia en la pareja ha resultado una cuestión compleja de verificar. Mientras que algunos estudios han encontrado asociaciones significativas entre la necesidad de controlar y el uso de la violencia contra la pareja^34^^,^^35^, otros han cuestionado la conceptualización tradicional del control como un constructo específico de género, o han señalado como disparadores de la violencia la existencia de múltiples motivantes adicionales al deseo de controlar^36^^,^^37^. 

En síntesis: la teorización feminista sobre la violencia de pareja descansa sobre tres conceptos centrales: patriarcado, género y poder. Con este enfoque, el énfasis tiende a ponerse en un nivel estructural, lo que lleva a percibir la eliminación de la violencia de pareja como una tarea inmensamente compleja: “*to end violence, we must eliminate patriarchy-a daunting task*” [para acabar con la violencia habría que acabar con el patriarcado, una tarea desalentadora]^38^^),^.

### El enfoque de la violencia familiar

También desde la década de 1970, se desarrolló la perspectiva de la violencia familiar, que argumenta que la violencia de pareja debe ser entendida no como un problema derivado fundamentalmente del “patriarcado” ni como una cuestión básicamente de género, sino como una expresión más del conjunto de violencias que ocurren al interior de la familia, como la violencia de los padres hacia los hijos, la violencia entre hermanos y hermanas, y otras semejantes, y que todas ellas se deben, en principio, a la “tolerancia cultural” que existe hacia estas formas de violencia^22^. 

Desde este enfoque, se considera la violencia en la pareja íntima como un fenómeno complejo en el que influyen diversas variables sociales y psicológicas, y no necesariamente las que se postulan desde el feminismo. La unidad de análisis es la familia y la clave para entender la violencia en la pareja es comprender qué lleva a los miembros de la familia a recurrir a la violencia como medio para resolver conflictos. El enfoque de la violencia familiar no teoriza mucho a nivel estructural y se centra en explorar el papel de las variables individuales y, en todo caso, del grupo al que pertenecen las personas estudiadas, argumentando -en ocasiones en completa oposición al enfoque feminista- que la desigualdad de género es muchas veces la consecuencia y no la causa de la violencia de pareja^39^^,^^40^. Desde esta perspectiva, se han desarrollado múltiples teorías, incluidas, además de las mencionadas más arriba, las teorías de sistemas, el modelo ecológico y la teoría de recursos, que examinan cómo la dinámica familiar y los factores sociales contribuyen a la violencia^27^.

Al enfoque de la violencia familiar se debe el desarrollo de la Escala Táctica de Conflictos, un instrumento que, al aplicarse en múltiples investigaciones, permitió el desarrollo de estudios comparativos^41^. Un argumento clave que marca una diferencia fundamental entre esta perspectiva y las teorías feministas es el planteamiento de que las mujeres son igualmente capaces que los hombres de ejercer violencia hacia sus parejas, aspecto que desde esta perspectiva se formuló como la simetría de género, abordándose con ello el problema de la bidireccionalidad de la violencia^42^^,^^43^.

Entre ambos enfoques se ha desarrollado un debate sumamente enriquecedor, en el que cada planteamiento ha aportado importantes evidencias en su favor^44^. En esta disputa ha sido central la cuestión de la bidireccionalidad de la violencia. Se ha acumulado creciente evidencia que muestra que las mujeres también agreden a sus parejas heterosexuales lo que pone en cuestión la narrativa dominante que supone a un hombre agresor y a una mujer víctima (si bien las consecuencias de la violencia física y sexual siempre son más graves para las mujeres que para los varones)^45^^,^^46^^,^^47^. Así, algunas autoras han comenzado a abogar por un enfoque más abierto a las evidencias, que incluya la cuestión de la reciprocidad y la violencia que también ejercen las mujeres hacia sus parejas^48^. En México también hay datos que sustentan que tanto varones como mujeres ejercen violencia contra sus parejas heterosexuales^49^^,^^50^^,^^51^.

Una contribución fundamental a este debate ha sido la propuesta de Johnson^52^, en el sentido de que es indispensable diferenciar entre los diversos tipos de violencia que pueden ocurrir al interior de las parejas heterosexuales. Johnson distingue tres tipos de violencia de pareja: terrorismo íntimo, resistencia violenta y violencia situacional de pareja^52^. Solo la primera, señala, está claramente asociada a la misoginia y al “tradicionalismo de género”, nociones ambas que podríamos explicar con un enfoque feminista. Las otras dos formas de violencia, sostiene, se explican mejor con enfoques más clínicos, psicológicos o incluso psicosociales. Fundamental en esta cuestión es que, de acuerdo al autor, las encuestas captan la resistencia violenta y la violencia situacional de pareja, mientras que el terrorismo íntimo (o sea, la violencia propiamente patriarcal), por su severidad, se aprecia más en las estadísticas de los refugios o policiales^53^^,^^54^. El enfoque de Johnson permite mirar más allá de las determinaciones estructurales y enfocarse sobre el tipo de vínculo que se establece. Esta contribución de Johnson ha resultado paradigmática en este campo de estudio y, como veremos más adelante, también se busca aplicarla, mediante adaptaciones específicas, al caso de la violencia en las parejas de la diversidad sexual.

## LIMITACIONES DE LOS PARADIGMAS DOMINANTES

Podríamos entonces resumir de la siguiente manera las principales limitaciones para abordar la violencia en la pareja íntima LGBTIQ+ desde los dos marcos teóricos dominantes:

1. El patriarcado como *causa última*: si bien el patriarcado sigue siendo un elemento relevante en la explicación de la violencia en la pareja íntima, es necesario un marco teórico más amplio e inclusivo, que abarque una variedad de explicaciones de esta para comprender y abordar plenamente este fenómeno^55^.
2. El predominio de un enfoque en la violencia masculina: es necesario superar el enfoque que se centra en la violencia de hombres hacia mujeres, pues ello no permite abordar ni explicar la violencia perpetrada por mujeres en relaciones heterosexuales ni la violencia en relaciones del mismo sexo^56^.
3. La visión rígida del poder en la pareja: no cabe asumir que el poder es unidireccional y rígido, con los hombres siempre en control o intentando mantener el control y la violencia en la pareja íntima como un instrumento masculino de control y de dominación^24^. Esto ignora las dinámicas de poder fluidas y relacionales que pueden existir en diferentes tipos de relaciones^57^.
4. Reducción de la agencia femenina: las teorías que enfatizan el papel de las estructuras patriarcales de dominación tienden a situar a las mujeres como “víctimas”, en posiciones de subordinación y vulnerabilidad^58^. Ello tiende a limitar la comprensión de cómo las mujeres pueden ejercer violencia por razones distintas a la autodefensa, lo que puede ser una simplificación excesiva de las motivaciones y circunstancias del ejercicio de violencia en la pareja íntima.
5. Heteronormatividad: no se suele abordar cómo la heteronormatividad y la homofobia afectan la dinámica de poder y la violencia en relaciones LGBTIQ+, dejando fuera de la discusión a estas comunidades. Es decir, ambas perspectivas tienden a centrarse en relaciones heterosexuales, ignorando las dinámicas únicas de las relaciones LGBTIQ+.
6. Discriminación hacia la comunidad LGBTIQ+: es necesario abordar cómo las experiencias de discriminación específicas de las personas LGBTIQ+ -como la homofobia internalizada y la discriminación- influyen en la violencia en la pareja íntima.
7. Modelos de intervención inadecuados para abordar la violencia en la pareja íntima LGBTIQ+: las intervenciones que están basadas en estas perspectivas pueden no ser efectivas para las parejas LGBTIQ+, ya que no consideran las experiencias y necesidades específicas de estas comunidades.
8. Visibilidad y estigmatización: las perspectivas feministas y de violencia familiar, en la medida en que no se formulan explícitamente para abarcar a la comunidad LGBTIQ+, pueden invisibilizar a las víctimas de violencia en la pareja LGBTIQ+ y perpetuar estigmas, dificultando el acceso a recursos y apoyo adecuado para esta población.

Estas limitaciones han tenido repercusiones importantes, no solo en términos de la capacidad explicativa de estos dos enfoques dominantes, sino también en su aplicación en esfuerzos específicos de prevención y atención de la violencia en la pareja íntima, ya que sistemáticamente han ignorado las condiciones específicas de la población LGBTIQ+. Hasta ahora, casi todos los esfuerzos de prevención y sensibilización en torno a la violencia en la pareja íntima derivados de estos marcos de comprensión han girado únicamente en torno a las parejas heterosexuales y cisgénero^59^. Del mismo modo, el enfoque feminista y de género convencional ha dado lugar a leyes, instituciones y servicios centrados en el paradigma del hombre agresor y la mujer agredida, dejando de lado y sin protección a los hombres y a la población de la diversidad sexual cuando son víctimas de violencia en la pareja íntima. Y ambos factores han repercutido en la noción más o menos generalizada, pero errónea, de que la violencia entre parejas del mismo sexo no es tan relevante como la violencia entre parejas heterosexuales^60^.

## DESARROLLOS TEÓRICOS Y EMPÍRICOS SOBRE VIOLENCIA EN PAREJAS DE LA DIVERSIDAD SEXUAL

La investigación sobre violencia en parejas de la diversidad sexual también se ha desarrollado por las dos vertientes que acabamos de examinar, es decir, por vía de los estudios teóricos (en donde el enfoque feminista y la teoría queer han tenido preeminencia) y por la vía de las investigaciones empíricas (en donde el enfoque de la violencia familiar ha dominado). Muchos aspectos de la violencia de pareja en los grupos LGBTIQ+ -como el papel de la dinámica de poder, la naturaleza cíclica del maltrato y la escalada del abuso a lo largo del tiempo- son similares entre las relaciones LGBTIQ+ y heterosexuales. Sin embargo, hay algunos aspectos de la violencia en la pareja íntima que son exclusivos de la experiencia LGBTIQ+^61^, y que requieren ser explícitamente incorporados en las propuestas explicativas.

Sin embargo, la investigación sobre la violencia de pareja íntima en poblaciones LGBTIQ+ aún requiere de un marco teórico integral, es decir, un marco que contemple tanto el nivel estructural como las variables de orden meso y microsocial. Los elementos claves a considerar incluyen la prevalencia y los aspectos únicos de la violencia en la pareja íntima en las relaciones LGBTIQ+, que difieren de los modelos heteronormativos^7^^,^^62^. Dado el carácter aún incipiente de los esfuerzos por desarrollar este marco teórico, optamos por tratar de diferenciar la literatura al respecto entre aquellos trabajos con orientación teórica más general, de aquellos otros que, con datos empíricos, se inscriben en la tradición de las teorías de alcance medio que mencionamos más arriba.

### Enfoques teóricos generales: el feminismo posestructural y la teoría queer

En la literatura existen varios llamados para conservar el poder de la teorización feminista y, al mismo tiempo, extenderlo para dar cuenta también de la violencia entre parejas LGBTIQ+^63^^,^^64^. También se ha señalado la importancia de usar el enfoque de la interseccionalidad en el caso de la violencia de las parejas LGBTIQ+. 

Como hemos venido señalando, los paradigmas tradicionales para explicar la violencia en la pareja íntima en las relaciones heterosexuales presentan limitaciones para comprender la violencia en las parejas LGBTIQ+^63^. En primer lugar, se ha señalado que las teorías prevalecientes sobre la violencia de pareja íntima en parejas LGBTIQ+ están limitadas por perspectivas heteronormativas y cisnormativas, lo que ha contribuido a una invisibilización de las experiencias de violencia en la pareja íntima LGBTIQ+^62^^,^^65^^,^^66^^,^^67^. 

El feminismo postestructural ha postulado la necesidad de planteamientos más flexibles e incluyentes para el abordaje y compresión de la violencia en la pareja íntima en parejas de la diversidad sexual^5^. Esta corriente señala que es necesario identificar las *mediaciones*, es decir, los factores que intervienen y moldean las relaciones entre la dimensión estructural y las variables micro y meso sociales. En la misma línea, el concepto de patriarcado ha sido criticado desde múltiples frentes, por su tendencia a englobar en una sola categoría a la diversidad de varones que existen y, concomitantemente, su falla para explicar por qué no todos los hombres ejercen violencia contra sus parejas^68^.

El feminismo postestructural retoma la propuesta de Foucault, para quien el poder no es solo el que existe entre el Estado y los individuos, sino que también es una fuerza que estructura las relaciones entre los individuos comunes y corrientes (docente-estudiante, personal de salud-paciente, sacerdote-feligrés, etc.). Este poder transversal organiza las relaciones entre los individuos de múltiples maneras, bien para reproducir estructuras de dominación (por ejemplo, la heteronormatividad), o bien para cuestionarlas (por ejemplo, el reto a la heteronorma por aquellos que asumen y exhiben una identidad que no se ajusta a los roles de género convencionales)^5^^,^^69^. 

Con base en ello, esta corriente propone que al deconstruir los binarismos convencionales (por ejemplo, masculino-femenino, heterosexual-homosexual, etc.) resulta evidente su inestabilidad, pues funcionan sobre la base de la subyugación del segundo término para definir el primero^63^. Como señalan Cannon y coautoras^63^, al deconstruir el par binario hombres-agresores/mujeres agredidas son más inteligibles los datos que muestran que las mujeres son tan capaces de iniciar y ejercer violencia contra los hombres como estos últimos.

Por otra parte, la teoría queer se enfoca en la crítica de las normas y categorías binarias de género y sexualidad. Argumenta que la identidad de género y la orientación sexual son construcciones sociales y culturales^70^. Esta teoría acepta que el género es una categoría central para el análisis institucional, interaccional e individual, pero postula que también lo es la sexualidad, dado que el heterosexismo y la homofobia están enraizados en las instituciones. La teoría queer sostiene que el binarismo heterosexual/homosexual funciona igual que los otros señalados más arriba: la subyugación del segundo es indispensable para definir al primero como la norma. La crítica queer se centra en la heteronormatividad, que sostiene, regula y oprime la diversidad sexual de las personas^71^. 

El feminismo posestructural y la teoría queer argumentan que la violencia que ejerce una mujer lesbiana contra su pareja (o cualquier persona de la diversidad sexual), no necesariamente es una extensión de la violencia de origen patriarcal teorizada por el feminismo de la segunda ola, sino que es básicamente una “táctica” más a su alcance. El conjunto de tácticas (por ejemplo, de violencia, de control, etc.) con las que se relaciona una pareja sería el objeto de estudio. Entenderlo así, se dice, nos evita caer en simplificaciones, como la noción de que la violencia entre parejas de la diversidad sexual se explica porque en estas hay una reproducción de los roles de género patriarcales^63^. Ambos enfoques (feminismo posestructural y teoría queer) postulan la necesidad de “hilar más fino” para evitar el determinismo rígido que supone atribuir al “patriarcado” el origen último de toda forma de violencia de pareja, y abrir la posibilidad de identificar los diversos contextos, trayectorias, circunstancias y otras variables que explican la violencia de pareja entre personas del mismo sexo^72^. 

En el contexto de estos desarrollos teóricos, algunas autoras postulan que el paradigma de género presenta ciertas limitaciones para dar cuenta de la violencia entre parejas del mismo sexo^73^. El enfoque, se señala, no considera las evidencias existentes acerca de la bidireccionalidad de la violencia en parejas hetero y de las diversas formas de violencia que puede haber entre parejas del mismo sexo^56^. Esta omisión, y la adscripción “obligatoria” al género, sostienen algunos autores, hace que sepamos muy poco acerca de las motivaciones de otros actores, no solamente varones, para ejercer violencia en el marco de relaciones de parejas LGBTIQ+^39^^,^^74^. El énfasis casi exclusivo en el género, señalan, hace que se pierda el papel que otras variables de corte psicosocial y el rol que otros contextos culturales más misóginos u homofóbicos pueden jugar en la explicación de la violencia en parejas tanto heteronormadas como no-heteronormadas^56^.

Por tanto, el enfoque de género exige nuevos desarrollos para explicar la dinámica de la violencia en las parejas de la diversidad sexual. Es preciso el aporte de otros enfoques para evitar los reduccionismos denunciados por el feminismo posestructural y la teoría queer, y superar el error que lleva a concluir que si hombres y mujeres ejercen por igual la violencia entonces la violencia de pareja no sería una cuestión de género^75^. Tal vez, las limitaciones de las explicaciones radican en que se pierden de vista los dos niveles de análisis en los que es preciso aplicar simultáneamente el enfoque de género: el interaccional y el estructural. Por vía del primero se pueden advertir en qué medida las personas “hacen género”, como lo propusieron West y Zimmerman^76^, al recurrir a la violencia de pareja; esto es, en qué medida dicha violencia es también constitutiva de (y no solo está constituida por) las relaciones de género. Por vía del segundo se puede apreciar en qué medida muchas de las conductas asociadas a la violencia de pareja, en cualquier tipo de parejas (heterosexuales y no heterosexuales) están condicionadas por las desigualdades estructurales de género. Ejemplos de esto último serían, entre otros, la desigual distribución del empleo (que puede dificultar que muchas personas agredidas abandonen la relación de pareja), o el riesgo real que puede significar, en una sociedad regida por valores sexistas, la amenaza de un integrante de una pareja de personas del mismo sexo de “poner en evidencia” a la otra ante otros familiares o compañeros de trabajo^63^^,^^77^.

Las perspectivas feministas recientes han ido más allá del patriarcado como causa *última* de la violencia en la pareja íntima, abogando por un enfoque interseccional que considera múltiples sistemas de opresión^55^^,^^78^. La interseccionalidad se ha mencionado como un marco importante para abordar tanto la violencia en la pareja íntima LGBTIQ+ como la violencia en parejas en relaciones de sexo opuesto. En la medida en que esta perspectiva teórica reconoce la existencia simultánea de múltiples categorías sociales como raza, etnia, género y orientación sexual que influyen en las experiencias individuales y, por tanto, en la existencia de múltiples sistemas entrelazados de opresión a nivel micro, este enfoque permite una mirada más comprehensiva de las condiciones particulares frente a la violencia en la pareja íntima de la diversidad sexual macro^66^.

### Teorías de alcance medio

Como ya señalamos, otro resultado de nuestra revisión se centró en la literatura que contribuye a la producción de teorías de alcance medio. El término fue desarrollado por Merton^105^ en oposición a las teorías generales o totalizadoras (que intentan explicar toda la realidad social) y a las hipótesis empíricas de corto alcance. Para Merton, las teorías de alcance medio debían estar basadas en evidencia empírica y ser lo suficientemente generales como para aplicarse a diferentes contextos, pero sin pretender explicar toda la sociedad en su conjunto.

La caracterización, más o menos rígida, de uno de los integrantes de la pareja como agresor (usualmente, el hombre) y a la otra persona como víctima (usualmente, la mujer), proviene en parte también, de acuerdo a algunos autores, del hecho de que muchas de las encuestas están diseñadas para medir el problema solo en esa dirección, es decir, que no exploran la violencia que pueden estar ejerciendo las mujeres y, por tanto, no producen datos en ese sentido^72^. Ello impide, por una parte, visualizar la posible bidireccionalidad de la violencia de pareja, pero, además, ha llevado a encasillar a las mujeres en la posición de víctimas^79^^,^^80^.

La investigación empírica muestra que un amplio número de variables que se asocian a la violencia entre parejas heterosexuales también se asocian a la violencia entre parejas de gay y lesbianas, tales como una serie de características sociodemográficas (edad, nivel educativo, nivel socioeconómico, contexto urbano o rural), experiencias de violencias en la infancia, la dinámica de poder en la relación, etc., así como los desbalances de poder en la pareja, el deseo de controlar a la otra persona y las inseguridades subyacentes^7^^,^^81^^,^^82^. 

Otros estudios admiten que, si bien la violencia en ambos tipos de pareja puede ser muy similar, entre las parejas no heterosexuales existen características específicas (por ejemplo, en ocasiones prevalencias superiores a las que se observan en las parejas heterosexuales, mayor letalidad y diferentes cursos de acción ante el problema) y que, por lo mismo, existe una serie de variables específicas de la población no heterosexual que explican estas características^83^. 

Diversos autores señalan la necesidad de incorporar en los marcos explicativos aquellos aspectos únicos en las experiencias de personas LGBTIQ+ que pueden afectar su vulnerabilidad frente a la violencia en la pareja íntima, tales como el estrés de las minorías (resultante de la homofobia experimentada e interiorizada), la homofobia, la homonegatividad, la “salida del closet” (revelación de la identidad u orientación sexual), el estigma y la discriminación, que no se abordan en los enfoques convencionales de la violencia en la pareja íntima^84^^,^^85^^,^^86^. También se ha documentado el papel de otras variables de particular relevancia para las relaciones entre personas del mismo sexo, tales como el estigma social al mostrarse abiertamente en público como parte de la comunidad LGBTIQ+^83^^,^^87^.

En efecto, un factor específico de la violencia en la pareja íntima en la población LGBTIQ+ es la homofobia internalizada^88^. Esta se asocia a características como baja autoestima, autoconcepto negativo y conflictos internos. Un autoconcepto negativo puede incrementar el riesgo de ser víctimas de violencia en la pareja íntima ya que las personas tienden a tolerar el abuso debido a su identidad sexual y, al mismo tiempo, facilita las posibilidades de convertirse en la persona agresora, en la medida en que estas personas pueden proyectar sus sentimientos negativos hacia sus parejas y cometer actos violentos contra ellas^59^. 

Estrechamente asociado a ello, diversos autores señalan que la violencia en la pareja íntima se asocia a múltiples problemas de salud mental como la depresión, el trastorno de estrés postraumático y trastornos por consumo de sustancias, padecimientos que, por supuesto, no son exclusivos de las personas de la diversidad sexual, pero que en el caso de las poblaciones vulnerables (como las personas LGBTIQ+) pueden resultar acentuados por la confluencia de diversos problemas de salud^89^.

Desde un enfoque de género, otros estudios se han centrado en la cuestión de las masculinidades (hegemónica y subalternas), para explicar la violencia de pareja entre varones. Se ha encontrado, por ejemplo, que también entre hombres gay existen diversas masculinidades jerarquizadas, y que tanto la heteronormatividad como la homofobia prevalecen en diversos niveles en la interacción entre parejas gay y en sus reacciones ante la violencia de pareja^90^. Es decir, cabe un análisis de género siempre que se retome el enfoque de las masculinidades, sin ignorar las controversias en dicho campo^91^^,^^92^. Se ha encontrado, que, tanto en parejas gay como lesbianas, al aplicar un test que mide índices de masculinidad, aquellas con índices más elevados tienden a tener mayores niveles de violencia^93^^,^^94^.

En esta misma línea, una corriente importante de estudios empíricos para explicar la violencia entre parejas del mismo sexo es la que se apoya en la teoría del estrés de las minorías (*minority stress*), propuesta por Meyer^95^ y que se refiere al conjunto de experiencias estresantes que se derivan del hecho de pertenecer a una minoría estigmatizada y marginada y desde la cual se puede ser objeto de abierta discriminación o, en el otro extremo, de microagresiones de diverso tipo que impactan igualmente en las personas^96^. Diversas investigaciones han mostrado que existe una correlación significativa entre este tipo de estrés y mucha de la violencia entre parejas del mismo sexo, al igual que factores como la homofobia internalizada, el grado en que los integrantes de una pareja del mismo sexo han salido del clóset, el grado de estigmatización que se percibe y las diversas formas de discriminación relacionadas con la orientación sexual y la identidad de género^7^^,^^97^. 

La investigación con base en la teoría del estrés de las minorías sexuales muestra que los estresores internalizados (homofobia, salida del clóset y conciencia del estigma) se asocian con mayores tasas de violencia interpersonal, mientras que los estresores externos (como las experiencias concretas de violencia, la discriminación y el acoso) no muestran correlación tan clara con la violencia de pareja^7^. Una forma particular de abuso se refiere al llamado “abuso de identidad” (*identity abuse*), que

…se refiere a las formas en que los perpetradores de violencia de pareja pueden emplear discriminación social heterosexista, homofóbica, bifóbica y transfóbica contra sus parejas sexuales y de minorías de género, socavando y devaluando así su identidad sexual o de género ya marginada. [Traducción libre de: *This form of violence refers to the ways in which IPV perpetrators may employ heterosexist, homophobic, biphobic, and transphobic societal discrimination against their sexual and gender minority partners, thus undermining and devaluing their already marginalized gender or sexual identity*].^98^

Finalmente, identificamos la publicación de dos trabajos muy recientes que contribuyen al desarrollo teórico y empírico de la investigación sobre violencia entre parejas de la diversidad sexual desde perspectivas novedosas. El primero de ellos aplica la teoría de los ensamblajes sociales al problema en cuestión^99^. Su premisa central es que lo que ocurre en las relaciones de pareja en las que hay abuso o violencia no se puede entender solo a partir del análisis de las identidades de los participantes o a través de un mero examen de las relaciones de poder jerárquicas. Con base en Deleuze y Guattari^100^ y mediante entrevistas en profundidad, la autora propone que es posible conceptualizar las dinámicas de abuso y violencia como efectos de diferentes elementos y relaciones afectivas que coinciden en un ensamblaje relacional abusivo. En sus propias palabras:

…en lugar de señalar las “causas” individuales o socioculturales de la violencia, analizo cómo el mantenimiento y la disputa de la violencia y el abuso emergen a través de nudos violentos de múltiples elementos humanos y no humanos (cuerpos, cosas, normas, ideas, instituciones sociales y constructos), todos los cuales tienen capacidades afectivas que se unen en relaciones abusivas, amplificando y/o disminuyendo los efectos de unos sobre otros. [Traducción libre de: *Instead of pointing back to individual or sociocultural “causes” of violence, I analyze how the maintenance and contestation of violence and abuse emerge through violent entanglements of manifold human and nonhuman elements (bodies, things, norms, ideas, social institutions, and constructs), which all have affective capacities that come together in abusive relationships, amplifying and/or diminishing one another’s effects*].^99^

El uso de categorías como ensamblajes rizomáticos, que “conectan cuerpos, acciones, cosas, afectos, discursos e ideas en muy diversas maneras [assemblages are rhizomatic: they connect bodies, actions, things, affects, discourses, and ideas in many different ways]”^99^, y nudos, constituye una ruta prometedora en el desarrollo de nueva investigación en esta materia.

El otro trabajo propone nuevas extensiones teóricas a la tipología de Johnson que mencionamos más arriba^54^, con el fin de hacer visible y superar la cis-heteronormatividad implícita en este modelo y así aprovechar sus ventajas para estudiar la violencia en parejas de la diversidad sexual^101^. Los autores señalan que, además de las tácticas comunes de abuso, en las parejas LGBTIQ+ se presentan otras que son específicas de esas comunidades. Por ejemplo, deliberadamente llamar por el género equivocado (“*misgender*”) a la otra persona, limitar su capacidad de presentarse en su identidad verdadera, o llamar la atención (con frecuencia a través de violencia física o sexual) hacia las partes del cuerpo que causan disforia (tocando los genitales, dañando partes que se están recuperando de cirugía, etc.). Proponen, en consecuencia, dos “extensiones teóricas” al modelo de Johnson: por una parte, cuestionar la rigidez entre los diferentes tipos de poder que propone Johnson (control coercitivo, violencia situacional, etc.) y pensarlas como procesos más fluidos. Y, por otra parte, reconceptualizar la noción misma de “poder” no solo como patriarcal, sino también a modo de desenmascarar su carácter cis-heteronormativo, y convertirla, así, en una categoría multidimensional sensible a las particularidades de las relaciones de las comunidades LGBTIQ+. Ello, señalan, permitirá dar cuenta de avances empíricos ya reportados como el estudio del “abuso de identidad”, que consiste en amenazar con “sacar del clóset” a una pareja, o impedir que busque ayuda en la comunidad queer, o forzar a la pareja a mostrar afecto en espacios donde se sienta con inseguridad para hacerlo. 

## CONCLUSIONES

### Sobre el estado de la cuestión

La narrativa dominante en la investigación sobre violencia en parejas heterosexuales presupone a un hombre agresor y a una mujer víctima, y fundamenta su explicación en el carácter patriarcal de la sociedad y en los desbalances de género en el poder de las partes. Sin embargo, al paso del tiempo ha quedado claro que esta teorización es insuficiente para dar cuenta de la violencia tanto en parejas heterosexuales como en parejas de la diversidad sexual. 

En este trabajo hemos explorado las teorías sociológicas existentes sobre los principales desarrollos que buscan dar cuenta de la violencia entre parejas LGBTIQ+ desde una perspectiva sociológica. Mostramos que, en el caso de la violencia en parejas heterosexuales, dos escuelas predominan en el ámbito científico: la perspectiva feminista y el enfoque de la violencia familiar. La primera propone que la violencia de pareja contra las mujeres debe entenderse como una más de las múltiples formas de violencia que sufren las mujeres (en el trabajo, la escuela, la calle, etc.) debido a las relaciones desiguales entre hombres y mujeres. Y que ello debe hacerse desde la perspectiva de la interseccionalidad. La segunda, en cambio, postula que la violencia que sufren las mujeres en la pareja debe entenderse como una forma más de las múltiples formas de violencia intrafamiliar que existen (de pareja, de padres a hijos, entre los hijos, de hijos a padres, etc.). Y cuestiona la pertinencia del enfoque de género para esta problemática. Ambas presentan desarrollos teóricos e investigaciones empíricas importantes. Mostramos que el modelo de Johnson, que enfatiza la necesidad de diferenciar diversos tipos de violencia de pareja (algunos mediados por cuestiones de género, otros no), es una de las propuestas más exitosas y prometedoras que permitiría articular las dos escuelas pues, al parecer, cada una estaría enfocándose a problemas diferentes.

En el caso de la violencia de parejas de la diversidad sexual, señalamos que los desarrollos teóricos más relevantes vienen del feminismo posestructural y de la teoría queer. Desde estos enfoques se propone recuperar la perspectiva de Foucault, que incluye las relaciones de poder no solo entre el Estado y los individuos, sino entre los individuos de diversa índole. Con esta perspectiva se abre la posibilidad de estudiar las dinámicas de poder que, enmarcadas en una sociedad patriarcal, homófoba y sexista, son específicas de las parejas de la diversidad sexual. 

Identificamos diversos obstáculos epistemológicos que han dificultado el avance de esta agenda de investigación, que incluyen, entre otros, el predominio de diseños de encuesta centrados en las relaciones heterosexuales y el temor de generar más animadversión contra las personas de la diversidad sexual. Mostramos que se ha constatado a nivel empírico, en primer lugar, que casi las mismas variables que caracterizan la violencia en parejas heterosexuales también funcionan como variables explicativas en la violencia entre parejas de la comunidad LGBTIQ+. Pero, en segundo lugar, que existen otra serie de variables, procesos y dinámicas que son específicos de esta última. El estrés de las minorías, la homofobia internalizada, las experiencias de violencia, discriminación y acoso figuran entre los varios factores asociados a las parejas no-heterosexuales. En esta revisión también señalamos la importancia (a partir de la enorme evidencia ya existente) de explorar la violencia ejercida (o perpetrada) y no exclusivamente la violencia recibida (o victimización). Ello permite una comprensión más integral de la dinámica del problema.

Y, finalmente, identificamos dos desarrollos recientes, muy incipientes aún, pero promisorios para el avance de la teorización sobre la violencia entre parejas de la diversidad sexual. Por una parte, los estudios centrados en la teoría de los ensamblajes sociales y, por otra, las propuestas de ampliación conceptual del modelo de Johnson con miras a superar el sesgo cis-heteronormativo que presenta. 

Al explorar el problema de la violencia interpersonal en parejas del mismo sexo, se corre el riesgo de poner junta a toda la población sexo-diversa, lo cual puede resultar contraproducente si cada subpoblación tiene su propia dinámica^102^. En la medida en que se siguen diversificando las identidades de género y las sexualidades, la investigación sobre la violencia de pareja en estas poblaciones ubicadas en los márgenes (y en “los márgenes de los márgenes”) debe diversificarse también^103^. Este señalamiento, sin embargo, plantea el desafío de atender a las características particulares de cada grupo de la diversidad sin caer en su *singularización* y estigmatización^4^.

### Sobre los retos en materia de investigación: hacia un marco explicativo

La heterogeneidad de personas y condiciones de la diversidad sexual plantea, en primer lugar, un desafío importante para cualquier intento de desarrollo de un marco explicativo de la violencia en la pareja íntima LGBTIQ+ que, a su vez, permita el desarrollo de políticas públicas y programas de intervención eficaces. La mayor parte de la investigación realizada sobre violencia de pareja en las poblaciones LGBTIQ+ se basa en muestras de gays y lesbianas supervivientes de la violencia en la pareja íntima; pero es muy probable que las personas bisexuales, trans y de género fluido sean aún más vulnerables a las tácticas de violencia en la pareja íntima específicas de la comunidad LGBTIQ+, así como también es posible que existan diferencias en las consecuencias de la violencia en la pareja íntima en función de la identidad de género y/o orientación sexual específicas^11^. El reto, entonces, es el desarrollo de un marco comprensivo que considere todos los factores de riesgo y los sistemas que pueden afectar a las personas, así como sus intersecciones.

En el esfuerzo de lograr explicar la violencia de pareja en la diversidad sexual es necesario tener presente no solo que se trata de parejas con otras características, diferentes a las de las parejas heterosexuales, sino que, además, al interior del grupo LGBTIQ+ sin duda también hay diferencias. Por ejemplo, cabe hipotetizar que la violencia entre parejas lesbianas, en ocasiones, puede diferir de la violencia entre parejas de hombres gay, en términos de sus dinámicas y motivaciones.

Es imperante que el desarrollo de los marcos teóricos que necesitamos para explicar la violencia entre parejas de la diversidad sexual, contemple como elemento central las distintas condiciones de las personas involucradas^55^, así como un mayor debate público para romper el silencio que rodea a la violencia en la pareja íntima en la comunidad LGBTIQ+^6^^,^^104^. Tales marcos deben conservar el *enfoque interseccional* que señalamos en la primera parte, es decir reconocer las diversas identidades dentro de la comunidad LGBTIQ+, incluyendo raza, identidad de género, orientación sexual, estatus socioeconómico y discapacidad. Esto ayuda a comprender cómo estas identidades que se entrecruzan influyen en las experiencias de violencia en la pareja íntima. Desde esta perspectiva se postula que los diferentes sistemas de opresión y discriminación se entrelazan y se superponen, creando experiencias únicas de múltiples formas de discriminación y desventaja para muchas personas. Además de ello, los marcos teóricos que necesitamos verían mejorada su capacidad explicativa si incluyeran los siguientes elementos:

- ***Teoría del estrés de las minorías***: reconocer que las personas LGBTIQ+ experimentan factores de estrés únicos, como la discriminación y la homofobia (o heterofobia) interiorizada (entre otros), que pueden contribuir a tasas más altas de violencia en la pareja íntima.
- ***Definiciones inclusivas***: definir claramente las variedades de la violencia en la pareja íntima de manera que abarque las diversas formas que puede adoptar en las relaciones LGBT, y no se reduzca solo al abuso físico, emocional, sexual y financiero.
- ***Barreras para la búsqueda de ayuda***: abordar las barreras específicas a las que se enfrentan las personas LGBTIQ+ al buscar ayuda frente a la violencia en la pareja íntima, como el miedo a la discriminación, la falta de servicios culturalmente competentes y la preocupación por salir del armario, en tanto que estos obstáculos pueden jugar un papel central en la perpetuación de la violencia.
- ***Marcos políticos y jurídicos***: analizar las políticas y las protecciones legales existentes para las personas LGBTIQ+ que sufren violencia en la pareja íntima, e identificar las lagunas que deben abordarse, en tanto que estas lagunas también pueden ser teorizadas como factores que contribuyen a la reproducción de la violencia.

La inclusión de estos elementos ayudará a crear marcos teóricos sociológicos más sólidos que aborden las necesidades y retos únicos a los que se enfrenta la investigación en materia de violencia de pareja en la comunidad LGBTIQ+.
