# Supplementary material for: Violencia entre parejas de la diversidad sexual y marcos teóricos explicativos: una agenda en desarrollo
Source: Salud Colect. 2025 Apr 15;21:e5558. [Article in Spanish] doi: 10.18294/sc.2025.5558 (PMC12306901; doi:10.18294/sc.2025.5558)
Supplement: Translation

# Translation: Intimate partner violence among sexual diversity couples and explanatory theoretical frameworks: A developing agenda

**Authors:** Roberto Castro, Irene Casique Rodríguez

**Affiliations:** 1 PhD in Medical Sociology. Researcher, Centro Regional de Investigaciones Multidisciplinarias, Universidad Nacional Autónoma de México, Cuernavaca, Mexico.. lucia.ariza@gmail.com; 2 PhD in Sociology. Senior Researcher, Centro Regional de Investigaciones Multidisciplinarias, Universidad Nacional Autónoma de México, Cuernavaca, Mexico. lima.natacha@hotmail.com

**Keywords:** Intimate Partner Violence, Sexual and Gender Minorities, Feminism, Social Construction of Gender

## Abstract

The aim of this article is to explore existing sociological theories on intimate partner violence among sexual diversity couples in order to generate an explanatory framework and identify the challenges faced by theorization in these populations. We take as a point of reference the feminist approach and the family violence approach-traditionally applied to heterosexual partner violence-to critically analyze the new theoretical proposals that have emerged to explain violence in sexually diverse couples, particularly poststructuralist feminism and queer theories. A growing development of theoretical frameworks and empirical research specifically focused on explaining intimate partner violence among LGBTIQ+ couples is observed. This article reflects on key sociological-feminist elements that should be incorporated into theoretical proposals addressing violence in non-heterosexual couples, and discusses some of the unresolved challenges that remain.

## INTRODUCTION

In recent years, significant progress has been made in research on intimate partner violence, which has been recognized as a human rights, political, social, and health issue with numerous consequences for individuals’ lives.^1^^,^^2^ Intimate partner violence is defined as:

“…any behavior within an intimate relationship that causes physical, sexual, or psychological harm, including acts of physical aggression, sexual coercion, psychological abuse, and controlling behaviors. This definition encompasses violence inflicted by current and former spouses and partners.”^3^

However, the vast majority of research efforts have focused on heterosexual couples, to the detriment of initiatives aimed at making this issue visible and addressing it when it occurs in sexually diverse couples.^4^ By “sexually diverse couples” (or LGBTQ+ couples), we refer to all those partnerships that do not fit the conventional description of “heterosexual” couples. This includes couples in which one or both partners have a non-heteronormative gender identity and/or sexual orientation. This is, evidently, a highly diverse group, which presents significant challenges for research.

The dominant narrative on intimate partner violence has, for many years, referred to heterosexual relationships, typically identifying a male perpetrator and a female victim.^5^ However, in recent years, the issue of violence in LGBTIQ+ couples has also begun to attract scientific interest, initially in the Anglo-American world,^6^ although it has gradually gained visibility in the research agendas of some Latin American countries such as Chile and Puerto Rico. This is due to growing evidence that violence in these couples occurs at equal or even higher rates than in heterosexual couples.^7^

In Latin America, interest in violence within LGBTIQ+ couples has also begun to emerge, though it remains marginal compared to the research (and public policy development) focused on heterosexual partner violence.^8^

The theorization regarding the nature, causes, and dynamics of violence within LGBTIQ+ couples is less consolidated than that developed in relation to heterosexual couples. Various authors argue that same-sex partner violence has not been studied as extensively, among other reasons, because it challenges the gender paradigm.^9^^,^^10^ Another reason is the ongoing stigmatization that same-sex couples face in many countries, along with the preference among some LGBTIQ+ groups to avoid drawing attention to this issue in order to prevent further stigmatization.^11^^,^^12^^,^^13^ This also makes it more difficult to turn to the police or support services when experiencing violence.^14^ Finally, myths such as the “lesbian utopia” (i.e., the idea of more egalitarian relationships between two women) have also been identified as a factor contributing to greater normalization of violent experiences within these couples and greater difficulty in recognizing them as such.^15^

The aim of this article is to explore existing sociological theories on violence in sexually diverse couples in order to develop an explanatory framework and identify the challenges faced by such theorization within these populations. To contextualize the topic, we begin by outlining the main sociological theoretical developments related to violence in heterosexual couples. From there, we focus on the critiques of these theories when applied to LGBTIQ+ populations, as well as the new theoretical proposals that have emerged to explain violence in sexually diverse couples. It is important to clarify that our intention is not to provide an exhaustive review of the state of the art nor to propose a new theory on the subject. Rather, we seek to critically examine the limitations of the theoretical approaches-particularly those grounded in sociological and feminist perspectives-that have traditionally prevailed in the study of violence in heterosexual couples, and the challenges these frameworks face in explaining violence within sexually diverse couples. In this regard, we aim to identify elements-particularly from poststructuralist and queer sociological approaches-that are essential to adopt or incorporate into more comprehensive theoretical frameworks capable of better accounting for the complexities of violence in these relationships.

## DOMINANT PARADIGMS

Intimate partner violence against women has been primarily studied from two different schools or approaches: the feminist approach and the family violence approach. These frameworks differ in a fundamental way: the feminist approach posits that violence against women is the most extreme manifestation of a broader structural pattern-that of women’s oppression under the patriarchal system-while the family violence approach holds that the overarching pattern underlying all forms of family violence is the cultural tolerance of interpersonal violence in our societies. Closely related to this difference in perspectives is another distinguishing feature of these approaches: the feminist approach offers a more refined theorization at the macro level, but precisely for this reason it often faces difficulties in operationalizing and empirically measuring key concepts such as “patriarchy,”^16^ “intersectionality,”^17^ or “culture of violence,”^18^ to name just a few. In contrast, the family violence approach prioritizes empirical research-based on the operationalization and measurement of variables-and from there develops middle-range theories.^19^ These include the “cycle of violence theory,”^20^ the application of exchange theory to explain partner violence,^21^ the “stress and social context theory,”^22^ and the “theory of violence as coercive control,”^23^ among many other examples. From this diversity of perspectives emerge two sometimes distinct scientific and political agendas, which we will examine in the following section.

### The feminist approach

Emerging from the extensive work carried out since the 1970s to make violence against women visible and a subject of critical concern, the feminist perspective developed as a set of theories, practices, and political movements focused on combating the oppression and discrimination of women. In the last quarter of the twentieth century, feminism posited that the ultimate cause of violence against women (in all its forms, including intimate partner violence) is patriarchy-that is, the social system based on the supremacy of men and masculinity over women and femininity.^24^^,^^25^ However, this ultimate cause falls short of being a sufficient explanation for a phenomenon as complex as the one under discussion.

Most academic research and public policy interventions addressing intimate partner violence, both in the Anglo-American world and in Latin America, began under the influence of the feminist paradigm emerging from activism and academia.^26^ Feminist theories provide a framework for explaining partner violence by emphasizing power dynamics based on gender and male domination.^27^ The dominant narrative within this perspective holds that men are the main aggressors and women the primary victims, and that gender inequality is the root cause of intimate partner violence and violence against women more broadly.^24^^,^^25^^,^^28^

In the early stages, during the 1970s, the issue was predominantly referred to using terms such as “wife battering,”^24^ “wife abuse,”^25^ or “wife beating.”^29^ However, as is characteristic of a field marked by power struggles,^30^ the various actors involved in the debate shifted in prominence and positioning, which led to changes in how the problem was named: from those earlier terms to “violence against women,” and later to “gender-based violence.” The latter term began to gain widespread use when United Nations agencies became central players in this discussion and the concept of gender was institutionalized.^31^^,^^32^

The gender approach posits that biological differences between the sexes are not the only factors influencing the inequalities and discrimination that people experience. This concept centers the analysis on how social, cultural, and political constructions of gender affect men and women in different ways, and how such inequalities may underlie the various forms of violence against women. From the feminist perspective, in any case, intimate partner violence against women is understood as one manifestation of a broader spectrum of violence that women experience in various settings, such as schools, workplaces, or public spaces. Therefore, it should be theorized as another expression of the inequality and oppression that women face.

In addition to the structural dimension, some strands of feminism have emphasized the need to focus analysis on the type of interpersonal relationship established within couples. On this level, violence in heterosexual relationships has been explained as a means used by men-among others-to exert control over women. This use of violence, in turn, draws its “legitimization” from patriarchal norms that underpin men’s prerogatives within a context of clear gender inequality.^25^^,^^26^^,^^33^

However, the role of control as a motivation for partner violence has proven to be a complex issue to verify. While some studies have found significant associations between the need for control and the use of violence against a partner,^34^^,^^35^ others have questioned the traditional conceptualization of control as a gender-specific construct, or have pointed to multiple additional motivations beyond the desire to control as triggers for violence.^36^^,^^37^

In summary, feminist theorization on intimate partner violence rests on three central concepts: patriarchy, gender, and power. From this perspective, emphasis tends to be placed on a structural level, which leads to the perception that eliminating intimate partner violence is an immensely complex task: “to end violence, we must eliminate patriarchy-a daunting task.”^38^

### The family violence approach

Also emerging in the 1970s, the family violence perspective argues that intimate partner violence should not be understood primarily as a problem stemming from “patriarchy” or essentially as a gender-based issue, but rather as one more expression of the broader set of violent behaviors that occur within families-such as parental violence against children, sibling violence, and similar forms-and that all of these are, in principle, due to the cultural tolerance of such forms of violence.^22^

From this perspective, intimate partner violence is seen as a complex phenomenon influenced by a variety of social and psychological variables-not necessarily those emphasized by feminism. The unit of analysis is the family, and the key to understanding partner violence lies in examining what leads family members to resort to violence as a means of resolving conflicts. The family violence approach does not focus heavily on structural theorization; instead, it emphasizes the role of individual-level variables and, in some cases, group characteristics of those studied. It argues-sometimes in direct opposition to feminist perspectives-that gender inequality is often a consequence rather than a cause of intimate partner violence.^39^^,^^40^ From this approach, numerous theories have been developed-including, in addition to those previously mentioned, systems theory, the ecological model, and resource theory-which examine how family dynamics and social factors contribute to violence.^27^

The development of the conflict tactics scale is attributed to the family violence perspective-an instrument that, through its application in numerous studies, enabled the advancement of comparative research.^41^ A key argument that marks a fundamental difference between this perspective and feminist theories is the claim that women are just as capable as men of perpetrating violence against their partners. This has been framed within this approach as gender symmetry, addressing the problem of bidirectionality in partner violence.^42^^,^^43^

An enriching debate has developed between these two approaches, with each contributing important evidence in its favor.^44^ A central issue in this dispute has been the question of bidirectionality in violence. Increasing evidence shows that women also perpetrate violence against their heterosexual partners, which challenges the dominant narrative that assumes a male aggressor and a female victim (even though the consequences of physical and sexual violence are consistently more severe for women than for men).^45^^,^^46^^,^^47^ As a result, some authors have begun to advocate for an evidence-based approach that is more open to the complexity of reciprocity and includes the violence that women also exert toward their partners.^48^ In Mexico, there is also data supporting the fact that both men and women perpetrate violence in heterosexual relationships.^49^^,^^50^^,^^51^

A key contribution to this debate has been Johnson’s proposal,^52^ which stresses the importance of differentiating between the various types of violence that may occur within heterosexual couples. Johnson identifies three forms of partner violence: intimate terrorism, violent resistance, and situational couple violence.^52^ Only the first, he argues, is clearly associated with misogyny and “gender traditionalism”-both notions that can be effectively explained through a feminist lens. The other two forms of violence, he maintains, are better understood through clinical, psychological, or even psychosocial approaches. Crucial to this discussion is Johnson’s observation that surveys tend to capture violent resistance and situational couple violence, while intimate terrorism (i.e., properly patriarchal violence), due to its severity, is more likely to appear in shelter or police statistics.^53^^,^^54^ Johnson’s framework allows for an analysis that goes beyond structural determinations and focuses on the type of relationship dynamics involved. His contribution has become paradigmatic in this field of study and, as we will see later, there have been efforts to apply it-with specific adaptations-to the case of violence in sexually diverse couples.

## LIMITATIONS OF DOMINANT PARADIGMS

We can summarize the main limitations in addressing intimate partner violence in LGBTIQ+ relationships from the two dominant theoretical frameworks as follows:

1. Patriarchy as the ultimate cause: While patriarchy remains a relevant element in explaining intimate partner violence, a broader and more inclusive theoretical framework is needed-one that incorporates a variety of explanations in order to fully understand and address this phenomenon.^55^
2. The predominance of a male violence approach: It is necessary to move beyond the framework that focuses solely on male violence against women, as it fails to account for or explain violence perpetrated by women in heterosexual relationships as well as violence in same-sex relationships.^56^
3. A rigid view of power within couples: It is not appropriate to assume that power is unidirectional and static, with men always in control or attempting to maintain control, and intimate partner violence as a male tool of control and domination.^24^ This perspective overlooks the fluid and relational dynamics of power that can exist in different types of relationships.^57^
4. Reduction of female agency: Theories that emphasize the role of patriarchal structures of domination tend to portray women as “victims,” situated in positions of subordination and vulnerability.^58^ This can limit the understanding of how women may exercise violence for reasons other than self-defense, potentially oversimplifying the motivations and circumstances involved in intimate partner violence.
5. Heteronormativity: These frameworks often fail to address how heteronormativity and homophobia affect power dynamics and violence in LGBTIQ+ relationships, thereby excluding these communities from the discussion. In other words, both perspectives tend to focus on heterosexual relationships, ignoring the unique dynamics of LGBTIQ+ partnerships.
6. Discrimination against the LGBTIQ+ community: It is essential to address how experiences of discrimination specific to LGBTIQ+ individuals-such as internalized homophobia and societal discrimination-influence intimate partner violence.
7. Inadequate intervention models to address LGBTIQ+ intimate partner violence: Interventions based on these paradigms may not be effective for LGBTIQ+ couples, as they often fail to take into account the specific experiences and needs of these communities.
8. Invisibility and stigmatization: Since feminist and family violence perspectives are not explicitly designed to include the LGBTIQ+ community, they can render LGBTIQ+ victims of intimate partner violence invisible and perpetuate stigma, thus hindering access to adequate resources and support for this population.

These limitations have had significant consequences, not only in terms of the explanatory power of these two dominant approaches, but also in their application to specific efforts aimed at preventing and addressing intimate partner violence, as they have systematically ignored the specific conditions of the LGBTIQ+ population. To date, nearly all prevention and awareness initiatives related to intimate partner violence developed from these frameworks have focused exclusively on heterosexual and cisgender couples.^59^ Similarly, the conventional feminist and gender-based approach has informed laws, institutions, and services based on the paradigm of the male aggressor and the female victim, leaving men and sexually diverse populations unprotected when they are victims of intimate partner violence. Both factors have contributed to the widespread-yet mistaken-notion that violence in same-sex relationships is less relevant than violence in heterosexual relationships.^60^

## THEORETICAL AND EMPIRICAL DEVELOPMENTS ON VIOLENCE IN SEXUALLY DIVERSE COUPLES

Research on violence in sexually diverse couples has also followed the two paths previously examined: on the one hand, theoretical studies (in which the feminist approach and queer theory have predominated), and on the other, empirical research (where the family violence approach has been more dominant). Many aspects of partner violence within LGBTIQ+ groups-such as power dynamics, the cyclical nature of abuse, and the escalation of violence over time-are similar in both LGBTIQ+ and heterosexual relationships. However, there are specific aspects of intimate partner violence that are unique to the LGBTIQ+ experience,^61^ and these must be explicitly incorporated into explanatory frameworks.

Nevertheless, research on intimate partner violence within LGBTIQ+ populations still lacks a comprehensive theoretical framework-one that integrates structural, meso-level, and micro-social variables. Key elements to consider include the prevalence and unique features of intimate partner violence in LGBTIQ+ relationships, which differ from heteronormative models.^7^^,^^62^ Given the still-emerging nature of efforts to develop such a framework, we propose to distinguish the existing literature between those works that take a broader theoretical orientation, and others that, based on empirical data, fall within the tradition of the middle-range theories previously discussed.

### General theoretical approaches: poststructuralist feminism and queer theory

Several contributions in the literature call for preserving the analytical power of feminist theorization while simultaneously expanding it to account for violence in LGBTIQ+ couples as well.^63^^,^^64^ The importance of using an intersectional approach in the study of intimate partner violence within LGBTIQ+ relationships has also been highlighted.

As previously noted, traditional paradigms for explaining intimate partner violence in heterosexual relationships present limitations when applied to LGBTIQ+ couples.^63^ First, it has been pointed out that prevailing theories on intimate partner violence in LGBTIQ+ couples are constrained by heteronormative and cisnormative perspectives, which has contributed to the invisibilization of LGBTIQ+ experiences of intimate partner violence.^62^^,^^65^^,^^66^^,^^67^

Poststructuralist feminism has advocated for more flexible and inclusive approaches to the study and understanding of intimate partner violence in sexually diverse couples.^5^ This perspective emphasizes the need to identify mediating factors-that is, those elements that intervene and shape the relationships between structural dimensions and micro- and meso-level variables. Along these lines, the concept of patriarchy has been widely critiqued for its tendency to group all men into a single category, and consequently, for its failure to explain why not all men engage in violence against their partners.^68^

Poststructuralist feminism draws on Foucault’s proposal that power is not limited to the relationship between the state and individuals, but is also a force that structures relationships among ordinary individuals (e.g., teacher-student, healthcare worker-patient, priest-parishioner). This transversal form of power organizes interpersonal relationships in multiple ways-either to reproduce structures of domination (such as heteronormativity) or to challenge them (for example, through the subversion of gender norms by those who assume and express nonconforming identities).^5^^,^^69^

Based on this perspective, poststructuralist feminism proposes that deconstructing conventional binaries (e.g., male-female, heterosexual-homosexual) reveals their instability, as these oppositions function by subordinating the second term in order to define the first.^63^ As Cannon and colleagues point out,^63^ deconstructing the binary of male-perpetrator/female-victim allows for a better understanding of empirical findings that show women are just as capable of initiating and committing violence against men as the reverse.

Queer theory, on the other hand, focuses on the critique of binary norms and categories related to gender and sexuality. It argues that gender identity and sexual orientation are social and cultural constructions.^70^ This theory acknowledges gender as a central category of institutional, interactional, and individual analysis, but also asserts the importance of sexuality, given that heterosexism and homophobia are deeply embedded in institutions. Queer theory contends that the heterosexual/homosexual binary functions like other binaries: the subjugation of the latter is essential to defining the former as the norm. Queer critique centers on heteronormativity, which it sees as regulating and oppressing sexual diversity.^71^

Poststructuralist feminism and queer theory argue that violence perpetrated by a lesbian woman against her partner (or any person within the sexually diverse community) is not necessarily an extension of patriarchal violence as theorized by second-wave feminism, but rather one among various “tactics” available to her. The set of tactics-such as violence, control, etc.-used in relational dynamics would thus become the object of study. Framing it this way, it is argued, helps avoid simplifications such as the notion that violence in sexually diverse couples occurs because they replicate patriarchal gender roles.^63^ Both approaches-poststructuralist feminism and queer theory-emphasize the need to adopt a more nuanced analysis in order to avoid the rigid determinism of attributing all forms of partner violence to “patriarchy,” and to instead open the possibility of identifying the different contexts, trajectories, circumstances, and other variables that explain partner violence in same-sex relationships.^72^

Within the context of these theoretical developments, some authors argue that the gender paradigm presents certain limitations for explaining violence in same-sex relationships.^73^ This perspective, it is noted, fails to incorporate existing evidence about the bidirectionality of violence in heterosexual couples and the various forms of violence that can occur in same-sex partnerships.^56^ This omission-and the “compulsory” adherence to gender categories-some authors claim, leaves us with very little understanding of the motivations of other actors, not only men, who perpetrate violence within LGBTIQ+ relationships.^39^^,^^74^ The almost exclusive emphasis on gender, they suggest, obscures the role of other psychosocial variables and the influence of cultural contexts-such as misogynistic or homophobic environments-in explaining violence in both heteronormative and non-heteronormative couples.^56^

Therefore, the gender approach requires further development to explain the dynamics of violence in sexually diverse couples. Contributions from other frameworks are needed to avoid the reductionisms highlighted by poststructuralist feminism and queer theory, and to overcome the mistaken conclusion that if both men and women perpetrate violence equally, then intimate partner violence is not a gender issue.^75^ Perhaps the limitations of existing explanations lie in the failure to simultaneously consider the two levels of analysis at which the gender approach must be applied: the interactional and the structural. Through the former, it becomes possible to observe the extent to which individuals “do gender,” as proposed by West and Zimmerman,^76^ when engaging in intimate partner violence-that is, the extent to which such violence is also constitutive of (and not only constituted by) gender relations. Through the latter, one can discern how many behaviors associated with partner violence, in all types of relationships (heterosexual and non-heterosexual), are shaped by structural gender inequalities. Examples of the latter include, among others, the unequal distribution of employment opportunities (which may hinder a survivor’s ability to leave a violent relationship), or the real risk posed, in a society governed by sexist values, when one partner in a same-sex relationship threatens to “out” the other to family members or coworkers.^63^^,^^77^

Recent feminist perspectives have moved beyond patriarchy as the ultimate cause of intimate partner violence, advocating instead for an intersectional approach that considers multiple systems of oppression.^55^^,^^78^ Intersectionality has been identified as an important framework for addressing both intimate partner violence in LGBTIQ+ relationships and in opposite-sex relationships. Since this theoretical perspective recognizes the simultaneous existence of multiple social categories-such as race, ethnicity, gender, and sexual orientation-that shape individual experiences, and therefore acknowledges the presence of multiple, interwoven systems of oppression at the micro level, it enables a more comprehensive understanding of the particular conditions surrounding intimate partner violence in sexually diverse relationships at the macro level.^66^

### Middle-range theories

As previously mentioned, another outcome of our review focused on literature contributing to the development of middle-range theories. The term was introduced by Merton^105^ in contrast to grand or totalizing theories (which attempt to explain all of social reality) and short-range empirical hypotheses. For Merton, middle-range theories should be grounded in empirical evidence and be general enough to apply across different contexts, without aiming to explain society as a whole.

The more or less rigid characterization of one partner as the aggressor (usually the man) and the other as the victim (usually the woman) also stems, according to some authors, from the fact that many surveys are designed to measure the problem only in that direction-meaning they do not explore violence perpetrated by women and, therefore, fail to generate data in that regard.^72^ This not only prevents the visualization of potential bidirectionality in partner violence, but also reinforces the framing of women exclusively as victims.^79^^,^^80^

Empirical research shows that a wide range of variables associated with violence in heterosexual couples are also associated with violence in gay and lesbian couples. These include a number of sociodemographic factors (such as age, educational level, socioeconomic status, and urban or rural context), experiences of violence during childhood, the power dynamics within the relationship, as well as power imbalances between partners, the desire to control the other person, and underlying insecurities.^7^^,^^81^^,^^82^

Other studies acknowledge that, although violence may be very similar in both types of relationships, non-heterosexual couples exhibit specific characteristics-for example, in some cases, higher prevalence rates than those observed in heterosexual couples, greater lethality, and different courses of action in response to the problem-which indicate that a set of variables specific to the non-heterosexual population help explain these differences.^83^

Various authors emphasize the need to incorporate into explanatory frameworks the unique aspects of LGBTIQ+ individuals’ experiences that may influence their vulnerability to intimate partner violence. These include minority stress (resulting from experienced and internalized homophobia), homophobia, homonegativity, “coming out” (disclosure of sexual orientation or gender identity), stigma, and discrimination-factors that are not typically addressed in conventional frameworks for understanding intimate partner violence.^84^^,^^85^^,^^86^ The literature has also documented the role of other variables particularly relevant to same-sex relationships, such as social stigma related to publicly expressing one’s identity as part of the LGBTIQ+ community.^83^^,^^87^

Indeed, a specific factor in intimate partner violence among the LGBTIQ+ population is internalized homophobia.^88^ This is associated with characteristics such as low self-esteem, negative self-concept, and internal conflict. A negative self-concept can increase the risk of becoming a victim of intimate partner violence, as individuals may tolerate abuse due to their sexual identity. At the same time, it may also facilitate becoming a perpetrator, insofar as individuals may project their negative feelings onto their partners and commit acts of violence against them.^59^

Closely related to this, several authors have pointed out that intimate partner violence is associated with multiple mental health issues such as depression, post-traumatic stress disorder, and substance use disorders. While these conditions are by no means exclusive to sexually diverse individuals, in the case of vulnerable populations-such as LGBTIQ+ people-they may be exacerbated by the convergence of multiple health-related challenges.^89^

From a gender-based perspective, other studies have focused on the issue of masculinities (both hegemonic and subordinate) to explain partner violence between men. Research has shown, for example, that among gay men there are also hierarchical forms of masculinity, and that heteronormativity and homophobia persist at various levels in the interactions between gay partners and in their responses to intimate partner violence.^90^ In other words, gender analysis remains relevant as long as it incorporates the framework of masculinities-while also acknowledging the ongoing controversies within that field.^91^^,^^92^ Studies have found that, in both gay and lesbian couples, those with higher masculinity index scores tend to report higher levels of violence.^93^^,^^94^

In the same line of inquiry, an important body of empirical research explaining violence in same-sex couples draws on minority stress theory, proposed by Meyer.^95^ This theory refers to the set of stress-inducing experiences that arise from belonging to a stigmatized and marginalized minority, which can expose individuals to overt discrimination or, at the other end of the spectrum, to various forms of microaggressions that nonetheless have significant impacts.^96^ Numerous studies have shown a significant correlation between this type of stress and many forms of violence in same-sex couples, as well as with factors such as internalized homophobia, the degree to which members of a same-sex couple have come out, the perceived level of stigma, and the different forms of discrimination related to sexual orientation and gender identity.^7^^,^^97^

Research based on minority stress theory indicates that internalized stressors-such as homophobia, coming out, and stigma consciousness-are associated with higher rates of interpersonal violence, whereas external stressors-such as concrete experiences of violence, discrimination, and harassment-do not show such a clear correlation with partner violence.^7^

A particular form of abuse identified in this context is known as “identity abuse,” which...

...refers to the ways in which IPV perpetrators may employ heterosexist, homophobic, biphobic, and transphobic societal discrimination against their sexual and gender minority partners, thus undermining and devaluing their already marginalized gender or sexual identity.^98^

Finally, we identified the publication of two very recent works that contribute to the theoretical and empirical development of research on violence in sexually diverse couples from novel perspectives. The first of these applies the theory of social assemblages to the issue at hand.^99^ Its central premise is that what happens in abusive or violent relationships cannot be understood solely through an analysis of the participants’ identities or by merely examining hierarchical power relations. Drawing on Deleuze and Guattari^100^ and using in-depth interviews, the author proposes that it is possible to conceptualize the dynamics of abuse and violence as effects of various elements and affective relations that come together in an abusive relational assemblage. In her own words:

Instead of pointing back to individual or sociocultural “causes” of violence, I analyze how the maintenance and contestation of violence and abuse emerge through violent entanglements of manifold human and nonhuman elements (bodies, things, norms, ideas, social institutions, and constructs), which all have affective capacities that come together in abusive relationships, amplifying and/or diminishing one another’s effects.^99^

The use of categories such as rhizomatic assemblages, which “connect bodies, actions, things, affects, discourses, and ideas in many different ways,”^99^ and knots, represents a promising avenue for the development of new research in this field.

The second work proposes new theoretical extensions to Johnson’s typology, which we discussed earlier,^54^ with the aim of making visible-and overcoming-the cis-heteronormativity implicit in that model, while preserving its analytical advantages for studying violence in sexually diverse couples.^101^ The authors argue that, in addition to common abusive tactics, LGBTIQ+ couples experience other forms of abuse that are specific to these communities. For example, deliberately misgendering the partner, limiting their ability to express their authentic identity, or drawing attention-often through physical or sexual violence-to body parts that cause gender dysphoria (e.g., touching genitalia, harming areas recovering from surgery, etc.). Accordingly, they propose two “theoretical extensions” to Johnson’s model: first, to challenge the rigidity of the distinctions between Johnson’s types of power (coercive control, situational violence, etc.), and instead conceive of them as more fluid processes; and second, to reconceptualize the very notion of “power” not only as patriarchal, but also to expose its cis-heteronormative character, transforming it into a multidimensional category that is sensitive to the specificities of relationships within LGBTIQ+ communities. This, they argue, would allow for the incorporation of existing empirical advances-such as the study of “identity abuse,” which may involve threatening to out a partner, preventing them from seeking help within the queer community, or forcing them to show affection in spaces where they feel unsafe doing so.

## CONCLUSIONS

### On the state of the question

The dominant narrative in research on violence in heterosexual couples presumes a male aggressor and a female victim, grounding its explanation in the patriarchal nature of society and in gendered power imbalances between partners. However, over time it has become clear that this theorization is insufficient to fully account for violence both in heterosexual couples and in sexually diverse relationships.

In this paper, we have explored existing sociological theories and the main developments that seek to explain violence in LGBTIQ+ couples from a sociological perspective. We have shown that, in the case of violence in heterosexual couples, two schools of thought dominate the scientific field: the feminist perspective and the family violence approach. The former argues that intimate partner violence against women should be understood as one of the many forms of violence women suffer (at work, at school, in public spaces, etc.) as a result of unequal relations between men and women-and that this should be analyzed from an intersectional perspective. The latter, by contrast, maintains that violence against women in intimate relationships should be understood as one among several forms of intrafamilial violence (between partners, from parents to children, between siblings, from children to parents, etc.), and it questions the relevance of the gender approach in addressing this issue.

Both perspectives have produced important theoretical developments and empirical research. We have shown that Johnson’s model-which emphasizes the need to differentiate between various types of partner violence (some mediated by gender, others not)-is one of the most promising and successful proposals, as it allows for a potential articulation between the two schools of thought. Indeed, it appears that each would be focusing on different problems.

In the case of violence in sexually diverse couples, we have pointed out that the most relevant theoretical developments stem from poststructuralist feminism and queer theory. These approaches advocate for reclaiming Foucault’s perspective, which includes power relations not only between the state and individuals, but also among individuals in various social contexts. This perspective opens up the possibility of studying power dynamics that-framed within a patriarchal, homophobic, and sexist society-are specific to sexually diverse couples.

We identified several epistemological obstacles that have hindered the advancement of this research agenda. These include, among others, the predominance of survey designs focused on heterosexual relationships and the fear of fueling further animosity against sexually diverse individuals. We have shown that, at the empirical level, first, many of the same variables that characterize violence in heterosexual couples also serve as explanatory factors for violence in LGBTIQ+ relationships. But secondly, there is a distinct set of variables, processes, and dynamics specific to the latter. Minority stress, internalized homophobia, and experiences of violence, discrimination, and harassment are among the factors particularly associated with non-heterosexual couples.

This review also highlights the importance-based on extensive existing evidence-of exploring not only experienced (or received) violence, but also perpetrated violence. This broader perspective enables a more comprehensive understanding of the dynamics of the problem.

Finally, we identified two recent and still incipient-yet promising-developments for advancing theorization on violence in sexually diverse couples. On the one hand, studies grounded in the theory of social assemblages; and on the other, conceptual extensions of Johnson’s model aimed at overcoming its cis-heteronormative bias.

When exploring the issue of interpersonal violence in same-sex couples, there is a risk of lumping together the entire sexually diverse population, which can be counterproductive if each subpopulation exhibits its own distinct dynamics.^102^ As gender identities and sexualities continue to diversify, research on partner violence within these populations-often located on the margins (and in the “margins of the margins”)-must also diversify.^103^ This observation, however, presents the challenge of addressing the specific characteristics of each group within the LGBTIQ+ spectrum without falling into singularization or stigmatization.^4^

### On research challenges: toward an explanatory framework

The heterogeneity of individuals and conditions within the sexually diverse population presents, first and foremost, a significant challenge to any attempt to develop an explanatory framework for intimate partner violence in LGBTIQ+ relationships-one that could, in turn, support the creation of effective public policies and intervention programs. Most research on intimate partner violence within LGBTIQ+ populations has been based on samples of gay and lesbian survivors of intimate partner violence. However, it is highly likely that bisexual, trans, and gender-fluid individuals are even more vulnerable to certain intimate partner violence tactics specific to the LGBTIQ+ community. It is also possible that the consequences of intimate partner violence may differ depending on specific gender identities and/or sexual orientations.^11^ The challenge, then, lies in developing a comprehensive framework that takes into account all risk factors and systemic influences affecting individuals, as well as their intersections.

In seeking to explain partner violence in sexually diverse relationships, it is important to recognize not only that these are couples with characteristics distinct from heterosexual relationships, but also that differences undoubtedly exist within the LGBTIQ+ group itself. For example, it can be hypothesized that intimate partner violence dynamics and motivations in lesbian couples may, in some cases, differ from those in gay male couples.

It is imperative that the development of the theoretical frameworks needed to explain violence in sexually diverse couples places the varying conditions of the individuals involved at its center.^55^ Additionally, greater public debate is needed to break the silence surrounding intimate partner violence within the LGBTIQ+ community.^6^^,^^104^ These frameworks must preserve the intersectional approach we outlined earlier-that is, they must acknowledge the diverse identities within the LGBTIQ+ community, including race, gender identity, sexual orientation, socioeconomic status, and disability. This helps to understand how these intersecting identities shape individuals’ experiences of intimate partner violence. From this perspective, it is posited that different systems of oppression and discrimination intersect and overlap, creating unique experiences of multiple forms of discrimination and disadvantage for many people.

Moreover, the explanatory power of the theoretical frameworks we need would be enhanced by the inclusion of the following elements:

- Minority stress theory: Recognize that LGBTIQ+ individuals experience unique stressors-such as discrimination and internalized homophobia (or heterophobia), among others-that may contribute to higher rates of intimate partner violence.
- Inclusive definitions: Clearly define the various forms of intimate partner violence in ways that encompass the diverse manifestations it can take in LGBTIQ+ relationships, rather than limiting it to physical, emotional, sexual, and financial abuse.
- Barriers to help-seeking: Address the specific barriers LGBTIQ+ individuals face when seeking help for intimate partner violence, such as fear of discrimination, lack of culturally competent services, and concerns about coming out, as these obstacles may play a central role in perpetuating the violence.
- Political and legal frameworks: Analyze existing policies and legal protections for LGBTIQ+ individuals experiencing intimate partner violence, and identify the gaps that need to be addressed-understanding these gaps as factors that may also contribute to the reproduction of violence.

The inclusion of these elements will help to build stronger sociological theoretical frameworks that address the unique needs and challenges faced by research on intimate partner violence within the LGBTIQ+ community.
